# Orexin-A Regulates Follicular Growth, Proliferation, Cell Cycle and Apoptosis in Mouse Primary Granulosa Cells via the AKT/ERK Signaling Pathway

**DOI:** 10.3390/molecules26185635

**Published:** 2021-09-16

**Authors:** Muhammad Safdar, Aixin Liang, Shahid Ali Rajput, Nasir Abbas, Muhammad Zubair, Aftab Shaukat, Aziz ur Rehman, Huma Jamil, Yan Guo, Farman Ullah, Liguo Yang

**Affiliations:** 1 Key Laboratory of Agricultural Animal Genetics, Breeding and Reproduction, Education Ministry of China, College of Animal Sciences and Technology, Huazhong Agricultural University, Wuhan 430070, China; safdar1126@webmail.hzau.edu.cn (M.S.); lax.pipi@mail.hzau.edu.cn (A.L.); aftabshaukat40@gmail.com (A.S.); guoyan@webmail.hzau.edu.cn (Y.G.); farman_aup@yahoo.com (F.U.); 2 Faculty of Veterinary and Animal Sciences, Muhammad Nawaz Shareef University of Agriculture, Multan 66000, Pakistan; dr.shahidali@hotmail.com; 3 Department of Animal Nutrition and Feed Sciences, College of Animal Science, South China Agricultural University, Guangzhou 510642, China; 4 Department of Animal Breeding & Genetics, PMAS-Arid Agriculture University, Rawalpindi 46300, Pakistan; nasirous121@gmail.com; 5 Key Laboratory of Agricultural Microbiology, Education Ministry of China, College of Veterinary Medicine, Huazhong Agricultural University, Wuhan 430070, China; zubair_durani@webmail.hzau.edu.cn; 6 Department of Pathobiology, University of Veterinary and Animal Sciences, Jhang 35200, Pakistan; aziz.rehman@uvas.edu.pk; 7 Department of Theriogenology, Faculty of Veterinary Science, University of Agriculture, Faisalabad 38000, Pakistan; drhjamil@hotmail.com

**Keywords:** orexin, granulosa cells, proliferation, apoptosis, cell cycle, AKT/ERK1/2 pathway

## Abstract

Granulosa cells (GCs) are essential for follicular growth, development, and atresia. The orexin-A (OXA) neuropeptide is widely involved in the regulation of various biological functions. OXA selectively binds to orexin receptor type 1 (OX1R) and mediates all its biological actions via OX1R. This study aimed to explore the expression of OXA and OX1R and their regulatory role in GCs proliferation, cell cycle progression, apoptosis, oocyte maturation, and underlying molecular mechanisms of these processes and elucidate its novel signaling pathway. Western blotting and RT-qPCR showed that OXA and OX1R were expressed during different developmental stages of GCs, and siRNA transfection successfully inhibited the expression of OX1R at the translational and transcriptional levels. Flow cytometry revealed that OX1R knockdown upregulated GCs apoptosis and triggered S-phase arrest in cell cycle progression. RT-qPCR and Western blotting showed significantly reduced expression of Bcl-2 and elevated expression of Bax, caspase-3, TNF-α, and P21 in OX1R-silenced GCs. Furthermore, the CCK-8 assay showed that knockdown of OX1R suppressed GCs proliferation by downregulating the expression of PCNA, a proliferation marker gene, at the translational and transcriptional levels. Western blotting revealed that knockdown of OX1R resulted in a considerable decrease of the phosphorylation level of the AKT and ERK1/2 proteins, indicating that the AKT/ERK1/2 pathway is involved in regulating GCs proliferation and apoptosis. In addition, OX1R silencing enhanced the mRNA expression of GDF9 and suppressed the mRNA expression of BMP15 in mouse GCs. Collectively, these results reveal a novel regulatory role of OXA in the development of GCs and folliculogenesis by regulating proliferation, apoptosis, and cell cycle progression. Therefore, OXA can be a promising therapeutic agent for female infertility.

## 1. Introduction

Mammalian folliculogenesis is a complex reproductive process that is regulated by autocrine [1], endocrine [2], and paracrine factors [3,4]. This process is involved in follicular development, atresia, and differentiation of granulosa cells, which are basic ovarian functional units. Granulosa cells secrete various substances, including sex hormones, cytokines, and growth factors, all of which are required to survive and develop follicles [5]. Furthermore, GCs can influence oocytes maturation via instructive paracrine and junctional interaction [6]. Therefore, follicular development or atresia mainly depends on the survival or death of GCs.

The degenerative processes or atresia in the ovary removes the vast majority of follicles, and a single or a few ovarian follicles undergo ovulation [7]. This degenerative process could be initiated by the apoptosis of GCs [8]. It is well-known that apoptosis and proliferation in GCs are associated with folliculogenesis, oogenesis, and atresia. Therefore, it is imperative to understand follicular growth, apoptosis, proliferation, steroidogenesis regulation, and underlying signaling pathways in GCs. In addition, identification of new molecules and factors associated with granulosa cell functions is very important for further understanding of folliculogenesis and its related complex molecular mechanisms. This understanding will eventually pave the way for the development of new molecular markers and the design of new therapeutic procedures for the treatment of ever-increasing fertility problems in mammals.

Orexin neuropeptides (orexin-A and -B) are primarily expressed in the hypothalamus and are produced from prepro-orexin [9,10]. Orexin-A (OXA) has a specific affinity to orexin receptor 1 (OX1R), while orexin-B has the same affinity to orexin receptors, OX1R and OX2R. OX1R selectively binds with orexin-A [11] and mediates all biological functions of OXA. Since its discovery in 1998, OXA involvement in feeding behavior, emotions, energy balance, sleep/wake cycle, and stress regulation has been reported [9,12,13]. Furthermore, the physiological effects of OXA in peripheral tissues, including the reproductive and endocrine systems, have been documented [14].

The expression and physiological role of orexin-A in several peripheral [14] and endocrine tissues such as the adrenal, pituitary, ovarian, and testicular tissues have been reported [15]. An interrelationship between orexins and the hypothalamic–pituitary–ovarian (HPO) system has been recognized [15]. As for the actions of orexins in the HPO axis, the elevated expression of OX1R, OX2R, and prepro-orexin during the proestrus evening in the hypothalamus and the pituitary gland of female cycling rats [16], ovaries of cats and dogs at all follicular stages [17] and the uterus of pigs has been documented [14]. Cycle-dependent expression of OX1R and OX2R in pigs, cats, and dogs indicates that the ovary is a major prospective site of orexinergic activity. Recent research reports have shown the potential involvement of OXA in the regulation of steroidogenic enzymes, secretion of progesterone and estrogen in the porcine uterus, and steroid hormones secretion during the early pregnancy and estrous cycle in pigs [18,19]. These limited studies suggest the novel physiological role of the OXA/OX1R signaling regulating the female reproductive system. However, the precise regulatory role of OXA in the ovarian granulosa cells growth, development and functions has not been studied.

In this study, we investigated the expression of orexin-A and OX1R in mouse primary GCs and the effects of OX1R silencing on GCs proliferation, cell cycle progression, apoptosis, oocyte maturation, and the underlying molecular mechanisms involved in these processes. Moreover, we explored the signaling pathway by which OXA regulates proliferation and apoptosis in mouse granulosa cells.

## 2. Materials and Methods

### 2.1. Chemicals and Antibodies

Fetal bovine serum (FBS), phosphate-buffered saline (PBS), and Dulbecco’s modified Eagle’s medium F12 (DMEM/F12) were provided by Hyclone, Inc. (Logan, UT, USA). The Lipofectamine^®^ RNAiMAX transfection reagent was bought from Invitrogen, Life Technology, Inc. (Carlsbad, CA, USA), and OPTIMEM^®^ was purchased from Gibco (USA). Total RNA Kit I was obtained from Omega Biotech (Norcross, GA, USA). The FastKing RT Kit was bought from Tiangin Biotech, Co., Ltd. (Tiangin, China). The BCA Protein Assay Kit was bought from Pierce (Rockford, IL, USA). The SYBR Green Master Mix was purchased from Qiagen (Hilden, Germany). The Annexin V FITC/PI apoptosis detection kit was purchased from KeyGen Biotech (Nanjing, China). Cell Counting Kit-8 was obtained from Donjindo Molecular Technologies, Inc. (Rockville, MD, USA). The OX1R antibody and the RIPA buffer were obtained from ThermoFisher Scientific (USA). Caspase-3, P21 were purchased from Santa Cruz Biotechnology, Inc. (Santa Cruz, CA, USA). Bax was obtained from Abcam (Cambridge, UK), while PCNA, Bcl-2, and TNF-α were purchased from San Ying Bio-Tech (Wuhan, China). Antibodies p-Akt and p-ERK1/2 were bought from CST (Danvers, MA, USA). GAPDH was purchased from XianZhi BioTech (Tiangin, China).

### 2.2. Experimental Mice

Three- and six-week-old experimental female mice (Kunming white strain) were purchased from Hubei Animal Breeding Center, Wuhan, China, and managed in a room with controlled humidity (65%) and temperature (64–79 °F) and the 12 h light/dark cycle. The mice were provided water and food round the clock. The experimental use of female mice in this study was performed in accordance with animal welfare standards approved by the Research Animal Ethics Committee of Huazhong Agricultural University (HZAUMO-2021–0016).

### 2.3. Cell Isolation and Culture

Female mice were killed by cervical dislocation, and the ovaries were collected under sterile conditions in fresh PBS. The isolated ovaries were punctured with needles, mechanically minced, and centrifuged at 1500 rpm for 5 min as previously described [20]. The supernatant was discarded, and pure GCs were seeded in a classic culture medium (DMEM/F12) enriched with 10% FBS and penicillin/streptomycin (100 U/mL). The cells were cultured in a humidified incubator (5% CO_2_, 95% O_2_) at 37 °C for 48 h. The identification and purity of GCs were checked by immunocytochemical staining of FSHR, a marker gene for granulosa cells.

### 2.4. Transfection with siRNA

Mouse primary granulosa cells were transfected with a pool of three siRNAs targeting OX1R and non-targeting control (NC) sequences using Lipofectamine^®^ RNAiMAX for 48 h. Both RNAiMAX and siRNAs were carefully diluted in OPTIMEM^®^ for granulosa cells transfection following the manufacturer’s guidelines. From a pool of three siRNAs, the siRNA with the highest silencing capacity (referred to as siOX1R in this manuscript) was selected for further experiments. The sequences of OX1R siRNAs are shown in Table 1.

### 2.5. Total RNA Extraction and the RT-qPCR Assay

RNA from mouse granulosa cells was extracted using Total RNA Kit I following the manufacturer’s instructions. After checking the quantity and purity using a Nanodrop 2000 Analyzer (Thermo Scientific, DE, USA), 1 µg of total RNA was used for cDNA synthesis using a FastKing RT Kit following the manufacturer’s guidelines. The expression of the OXA, OX1R, caspase-3, Bcl-2, P21, PCNA, BMP15, and GDNF9 genes was investigated using the SYBR Green Master Mix. RT-qPCR values were standardized with β-actin, and the calculation of expression fold changes of the target gene was performed using the 2^−∆∆Ct^ method. The sequences of specific primers for RT-qPCR are listed in Table 2.

### 2.6. Protein Extraction and Western Blot Assay

Total protein from siRNA-transfected mouse GCs was extracted using a RIPA buffer containing 1% protease inhibitor, 1% phenylmethanesulfonyl fluoride, and 1% phosphate buffer. A BCA protein assay kit was used to quantify total protein following the manufacturer’s instructions, and the samples were kept at –80 °C for subsequent use. Equal volumes of total protein per samples were separated using 12% SDS–PAGE and transferred to polyvinylidene difluoride membranes (Millipore, Billerica, MA, USA). The membranes were treated with 5% skimmed milk diluted in Tris-buffered saline for 2 h at room temperature and incubated overnight at 4 °C with specific antibodies; OX1R (1:300); caspase-3, P21 (1:500); Bax (1:1000); p-ERK1/2 (1:1000); Bcl-2, TNF-α, PCNA (1:1000); p-AKT (1:2000); GAPDH (1:1000); and HRP-labeled goat anti-rabbit or rabbit anti-goat antibodies (1:3000). The identification of target proteins in each sample was performed using enhanced chemiluminescence (Bio-Rad, USA) following the manufacturer’s instructions. Finally, Western blot analysis was performed using the Gel-Pro Analyzer version 6 (Media Cybernetics, Rockville, MD, USA) and ImageJ software, and the results were standardized with GAPDH.

### 2.7. Cell Proliferation Assay

For proliferation assessment, mouse GCs were transfected with siOX1R and NC and cultured in 96-well culture plates at 2 × 10^5^ cells per well for 48 h at 37 °C. The proliferation capacity of GCs was determined using Cell Counting Kit-8 according to the manufacturer’s protocol. The viability of cells was measured based on the quantity of a viable indicator dye, formazan. In each well, 100 mL of the CCK-8 solution was added to 10% culture media and incubated for 2 h in the dark at 37 °C. Then, the absorbance at the OD of 450 nm for each experimental sample was measured using a microplate reader (PerkinElmer, EnSpire, USA). This result was further confirmed by measuring the protein and mRNA expression levels of the proliferation marker gene, PCNA.

### 2.8. Apoptosis Assay

Mouse GCs apoptosis was assessed with flow cytometry using an apoptosis kit (Annexin V FITC/PI) following the manufacturer’s protocol. Mouse primary granulosa cells were transfected with siOX1R and NC for 48 h at 37 °C. The cells were lysed using trypsin, washed three times with PBS, and resuspended in 500 mL of the binding buffer. Then, the cells were stained with Annexin V FITC (5 µL) and PI (5 µL) in a dark chamber at 4 °C for 20 min. Finally, the samples were analyzed with flow cytometry using FACSVerse Calibur (BD Biosciences, Columbia, USA) according to the manufacturer’s instructions. The results of flow cytometry were verified by measuring the protein and mRNA expression levels of apoptosis-related genes.

### 2.9. Cell Cycle Assay

For the assessment of cell cycle progression, GCs were harvested using trypsin after siOX1R transfection for 48 h at 37 °C. The harvested cells were extensively washed with cold PBS three times and kept in 75% ethanol at 4 °C for 12 h. Then, the cells were washed with PBS three times and stained with the PI solution (400 mL) and RNase (100 mL) for 30 min under darkened conditions. FACSVerse Calibur (BD Biosciences, USA) was used to assess the cell cycle progression, and the ModFit LT software (version 4) was used to analyze the percentage of cells in each cell cycle phase. For each analysis, a minimum of 20,000 cells were analyzed.

### 2.10. Statistical Analysis

The SPSS (SPSS Inc., Chicago, IL) software was used for statistical analysis of the data obtained from three independent experiments. Student’s *t*-test was performed to determine significant differences between the treatment and control groups. One-way ANOVA followed by Tukey’s *t*-test was performed to find the significant difference between different groups. The significant difference was defined as *p* < 0.05. All the results are shown as the means ± SEM as depicted in graphs using GraphPad Prism 7.

## 3. Results

### 3.1. Orexin-A via OX1R Regulates Mouse GCs during Different Developmental Stages

**Figure 1:**
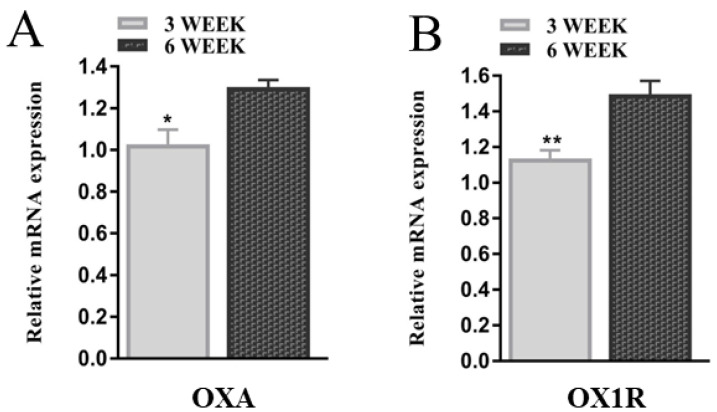
The expression of OXA and OX1R during different developmental stages of mouse GCs. (**A**,**B**) GCs of 3 week and 6 week-old female mice were cultured for 48 h, and total RNA was extracted. The mRNA expression of OXA and OX1R in mouse GCs of three- and six-week-old mice were detected using RT-qPCR. The expression values were normalized against β-actin and shown as the means ± SEM; * *p* < 0.05 and ** *p* < 0.01; GCs, granulosa cells; OXA, orexin-A; OX1R, orexin receptor type 1.

To explore the potential involvement of OXA and OX1R in different developmental stages of mouse granulosa cells, the mRNA expression levels of OXA and OX1R in GCs of the three- and six-week-old female mice were determined using RT-qPCR. The results showed that the mRNA expression levels of OXA and OX1R were significantly higher (* *p* < 0.05, Figure 1A; ** *p* < 0.01, Figure 1B, respectively) in the six-week-old compared to three-week-old female mice, indicating that the expression of OXA and OX1R is developmentally regulated.

### 3.2. The Expression of OX1R Was Efficiently Knocked down by Short Interfering RNA (siRNA) in Mouse GCs

**Figure 2:**
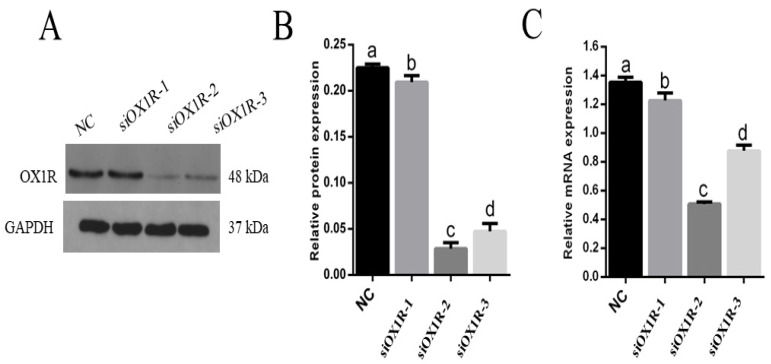
Effect of siRNAs on the expression of OX1R at the protein and mRNA levels in mouse GCs. (**A**–**C**) The protein and mRNA expression levels of OX1R were measured using Western blotting and RT-qPCR in siRNA-transfected mouse GCs. The values show the expression levels of OX1R relative to β-actin (RT-qPCR) and GAPDH (Western blotting). The data from three independent experiments were presented as the means ± SEM. Different superscripts ^a,b,c,d^ represent the level of significance between NC and siRNAs (*p* < 0.05). OX1R, orexin receptor 1; GC, granulosa cells; siOX1R, orexin receptor 1 siRNA; NC, negative control; siRNA, short interfering RNA.

To investigate the involvement of OXA in the development and regulation of mouse GCs functions, we used short interfering RNA (siRNA) to disrupt the expression of OX1R, a specific receptor of orexin-A, at the transcriptional and translational levels in mouse GCs. To enhance the efficiency of OX1R downregulation, we assessed a pool of three siRNAs targeting different regions of OX1R, namely siRNA-A, siRNA-B, and siRNA-C. After transfection for 48 h, Western blotting and RT-qPCR were performed to determine the transfection efficiency of OX1R siRNAs (siOX1R). The results showed that siRNAs transfection significantly inhibited the expression of OX1R in GCs at the protein and mRNA levels compared with the negative control. The Western blot and RT-qPCR analysis revealed that siRNA-B had the highest silencing capacity at the protein (0.2251 ± 0.022, *** *p* < 0.001; Figure 2A,B) and mRNA (0.5093 ± 0.07, *** *p* < 0.001; Figure 2C) levels. Therefore, siRNA-B was selected for further experiments to explore the novel functions and regulatory roles of orexin-A in mouse primary GCs.

### 3.3. Downregulation of OX1R Promoted Apoptosis in Mouse GCs

**Figure 3:**
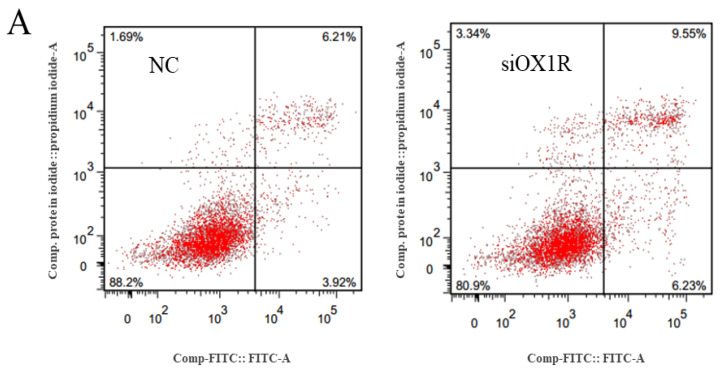

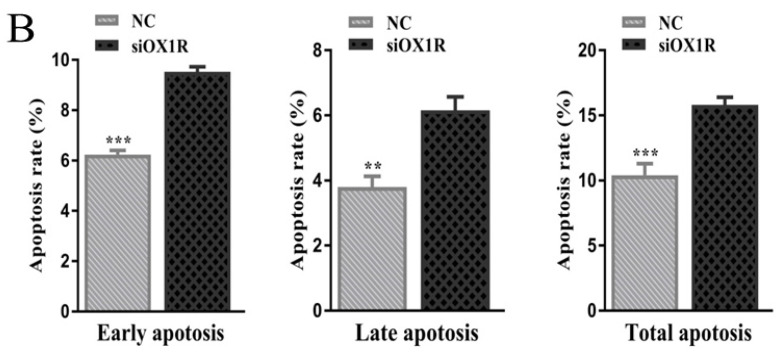
Downregulation of OX1R promoted apoptosis in mouse primary GCs. (**A**) Following the transfection with siOX1R and NC for 48 h, apoptosis in GCs was determined using flow cytometry. (**B**) The percentage of apoptosis was analyzed separately for early (lower right quadrants), late (upper right quadrants), and total apoptosis. The results are presented as the means ± SEM of three independent experiments. Different superscripts represent the level of significance between NC and siOX1R (** *p* < 0.01, *** *p* < 0.001). OX1R, orexin receptor type 1; NC, negative control; GCs, granulosa cells; siOX1R, orexin receptor type 1 siRNA.

For this study, Annexin V FITC/PI and flow cytometry were used to explore the role of OXA via OX1R in the regulation of mouse GCs apoptosis after transfection with siOX1R for 48 h. The results of flow cytometry showed that the apoptosis rate was significantly higher in siOX1R-transfected GCs groups (10.41 ± 0.50 vs 15.81 ± 0.33, *** *p* < 0.001) in comparison with the negative control GCs groups (Figure 3A,B). Downregulation of OX1R leads to the promotion of apoptosis in mouse GCs.

### 3.4. Knockdown of OX1R Altered the Expression of Apoptosis-Related Genes in Mouse GCs

**Figure 4:**
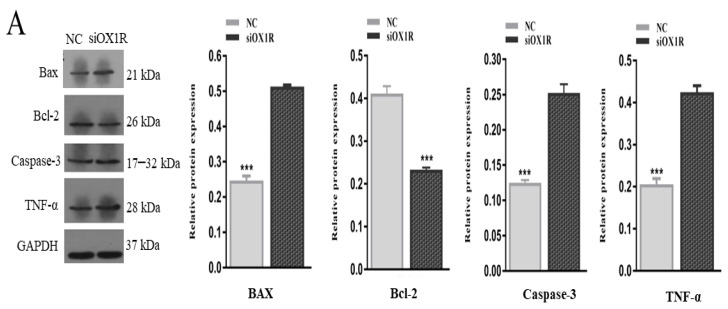

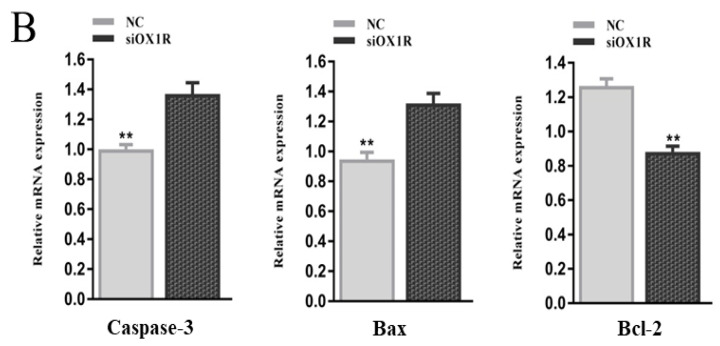
Knockdown of OX1R altered the expression of the key apoptotic regulator genes in mouse GCs. (**A**) Relative protein expression levels of Bax, caspase-3, Bcl-2, and TNF-α measured by Western blotting in mouse GCs after siOX1R transfection for 48 h. (**B**) Relative mRNA expression levels of Bax, caspase-3, and Bcl-2 detected by RT-qPCR in mouse GCs after siOX1R transfection for 48. The values show the expression levels of TNF-α, caspase-3, Bax, and Bcl-2 relative to β-actin (RT-qPCR) and GAPDH (Western blotting), and the data are shown as the means ± SEM of three independent experiments (** *p* < 0.01, *** *p* < 0.001). NC, negative control; OX1R, orexin receptor type 1; GCs, granulosa cells; siOX1R, orexin receptor type 1 siRNA.

To find out the underlying molecular mechanism of OXA-mediated apoptosis in mouse GCs, Western blot and RT-qPCR assays were performed to investigate the expression levels of the key apoptosis regulator genes. The knockdown of OX1R resulted in a significant reduction in the expression of Bcl-2 (anti-apoptotic factor) at the protein (*** *p* < 0.001, Figure 4A) and mRNA (** *p* < 0.01, Figure 4B) levels compared with negative control groups. The protein and mRNA expression levels of proapoptotic factors, caspase-3 (*** *p* < 0.001, Figure 4A; **p < 0.01, Figure 4B), Bax (*** *p* < 0.001, Figure 4A; ** *p* < 0.01, Figure 4B), were found significantly higher in siOX1R-transfected GCs groups compared with NC groups. Moreover, the protein expression of TNF-α (*** *p* < 0.001, Figure 4A) was also upregulated after siOX1R transfection of mouse GCs.

### 3.5. Downregulation of OX1R Induced S-Phase Arrest of the Cell Cycle in Mouse GCs

**Figure 5:**
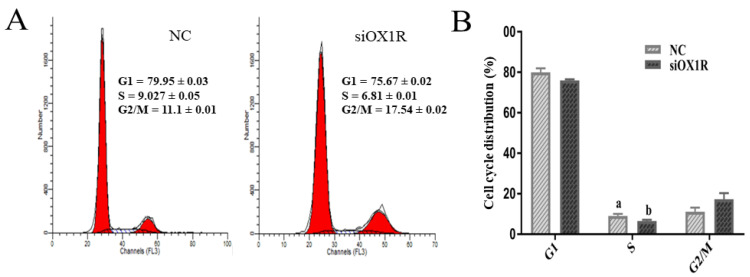

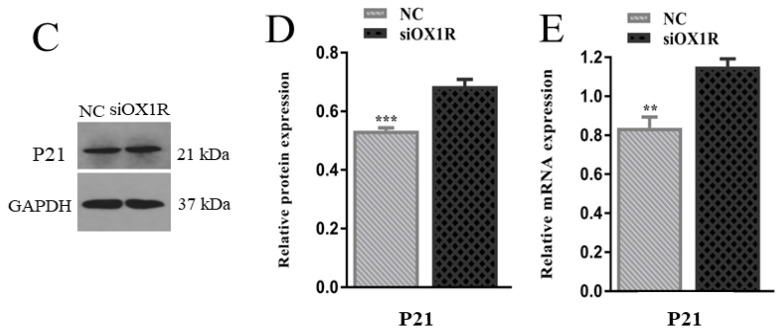
OX1R silencing induced S-phase arrest of the cell cycle in mouse GCs. (**A**) Measurement of the cell cycle progression using flow cytometry in mouse GCs after transfection with siOX1R for 48 h and (**B**) the cells distribution in different phases of the cell cycle. The significant difference (** *p* < 0.01) between different groups is shown with letters ‘a’ and ‘b’ within columns. (**C**–**E**) The expression of P21 at the mRNA and protein levels. The data are shown as the means ± SEM (** *p* < 0.01, *** *p* < 0.001). OX1R, orexin receptor type 1; NC, negative control; GC, granulosa cells; siOX1R, orexin receptor 1 siRNA; FACS, fluorescence-activated cell sorting.

To find out the influence of OXA on cell cycle progression of mGCs, the siOX1R-transfected cells were stained with PI/RNase, and the percentage of cells in each phase of the cell cycle were analyzed using flow cytometry. The results showed that siOX1R transfection induced S-phase arrest in the cell cycle of mouse GCs compared to the negative control (** *p* < 0.01, Figure 5A,B). To verify the cell cycle assay findings, we performed the transcriptional and translational analysis of the S-phase of cell cycle regulator marker gene, P21. RT-qPCR and Western blot assays showed the upregulated expression of P21 at the protein (*** *p* < 0.001, Figure 5C,D) and mRNA (** *p* < 0.01, Figure 5E) levels after siOX1R transfection for 48 h as compared to the negative control.

### 3.6. Knockdown of OX1R Inhibited Proliferation of Mouse GCs In Vitro

**Figure 6:**
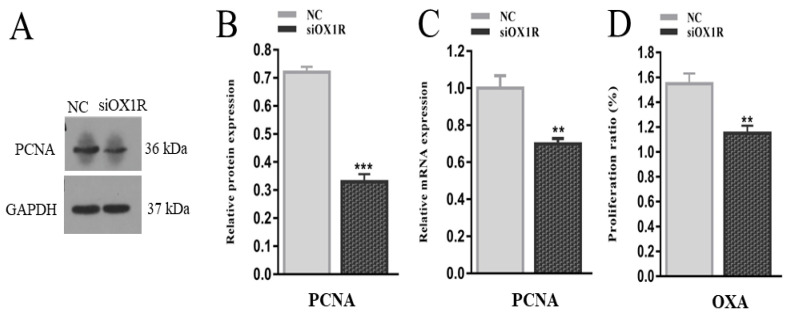
Knockdown of OX1R suppressed proliferation of mouse GCs. (**A**–**C**) The expression of the proliferation marker gene, PCNA, at the protein and mRNA levels was analyzed using Western blotting and RT-qPCR in mouse GCs after siOX1R and NC transfection for 48 h. (**D**) The proliferation rate of siOX1R-transfected GCs groups and the negative control groups were evaluated using the CCK-8 assay. The results are shown as the means ± SEM, and significant differences are indicated with error bars with asterisks (** *p* < 0.01 and *** *p* < 0.001). NC, Negative control; siOX1R, orexin receptor type 1 siRNA; OX1R, orexin receptor type 1; GCs, granulosa cells.

To find out the role of OXA in cell proliferation, following the siRNA-mediated downregulation of OX1R, the proliferation rate in mouse GCs was measured using Cell Counting Kit-8. The results revealed that OX1R silencing led to the suppression of proliferation in siOX1R-transfected GCs groups compared to the NC groups (*** *p* < 0.001, Figure 6C). Additionally, we verified this result by investigating the expression of the proliferation marker gene, PCNA, at the translational and transcriptional levels after the downregulation of OX1R in GCs. The Western blotting and RT-qPCR results showed downregulated expression of PCNA at the protein (*** *p* < 0.001, Figure 6A,B) and mRNA (** *p* < 0.01, Figure 6A,B) levels in siRNA-transfected GCs groups as compared to the NC groups.

### 3.7. Knockdown of OX1R Regulates Apoptosis and Proliferation through the AKT/ERK1/2 Signaling Pathway

**Figure 7:**
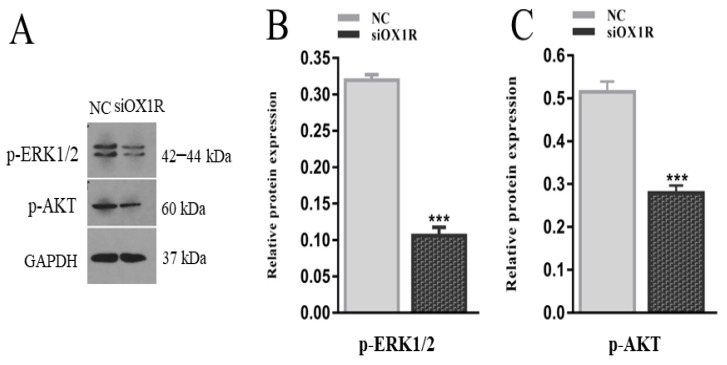
Effect of OX1R knockdown on the phosphorylation level of the AKT and ERK1/2 proteins in mouse GCs. (**A**–**C**) Mouse GCs were transfected with siOX1R for 48 h, and the protein was extracted for Western blot analysis of the phosphorylation level of the AKT and ERK1/2 proteins. The Western blot results were standardized with GAPDH and are presented as the means ± SEM. The significant differences between the siOX1R-transfected cells groups and the negative control group is indicated with error bars with asterisks (*** *p* < 0.001). OX1R, orexin receptor type 1; NC, negative control; GCs, granulosa cells; siOX1R, orexin receptor type 1 siRNA; AKT, Akt serine/threonine kinase; ERK, extracellular signal-regulated kinase.

To elucidate the novel signaling pathways by which OXA via OX1R regulates the proliferation and apoptosis in mouse GCs, we assessed the phosphorylation level of the AKT and ERK1/2 proteins by Western blotting following the siRNA-mediated downregulation of OX1R. The downregulation of OX1R significantly inhibited the phosphorylation level of the AKT and ERK1/2 proteins compared to the negative control groups (*** *p* < 0.001, Figure 7A–C. These results demonstrate that OXA promotes proliferation and suppresses apoptosis in mouse GCs by activating the AKT/ERK1/2 signaling pathway.

### 3.8. Knockdown of OXA Altered the mRNA Expression of Oocyte-Related Factors in Mouse GCs

**Figure 8:**
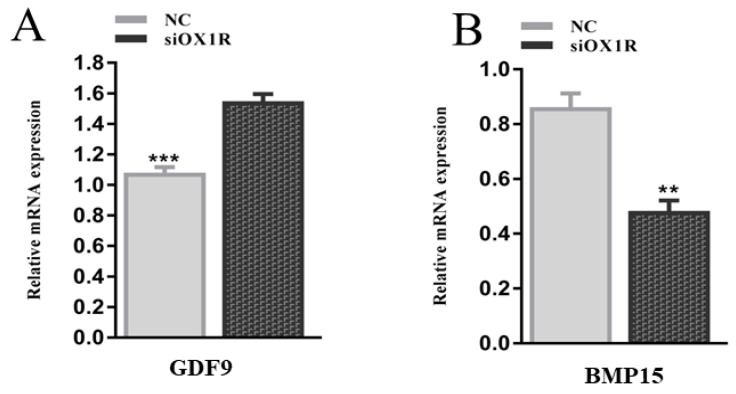
Effect of OX1R knockdown on the expression of oocyte-related genes in mouse GCs. (**A**,**B**) The mRNA expression of GDF9 and BMP15 was detected using RT-qPCR following siOX1R transfection for 48 h. The results are presented as the means ± SEM, and significant differences are indicated with error bars with asterisks (** *p* < 0.01, *** *p* < 0.001). NC, negative control; siOX1R, orexin receptor type 1 siRNA; OX1R, orexin receptor type 1; GC, granulosa cells; GDF9, growth differentiation factor 9; BMP15, bone morphogenetic protein 15.

To find out the regulatory role of OXA in the ovarian functions of female mice, an RT-qPCR assay was performed to quantify the mRNA expression levels of oocyte-related factors, the BMP15 (bone morphogenetic protein 15) and GDF9 (growth differentiation factor 9) genes. The results showed that siRNA-mediated downregulation of OX1R significantly promoted the mRNA expression level of GDF9 (*** *p* < 0.001, Figure 8A) and downregulated the mRNA expression level of BMP15 (** *p* < 0.01, Figure 8B). These results indicate the potential involvement of the OXA–OX1R signaling in intraovarian functions and oocyte maturation.

## 4. Discussion

Since the discovery of orexins, the involvement of orexin-A in the hypothalamic–pituitary–ovarian axis has been documented; however, its regulatory functions and molecular mechanisms in the female reproductive system have remained unclear. Therefore, we investigated the expression of OXA and OX1R at different developmental stages of GCs and explored their involvement in the regulation of proliferation, cell cycle progression, apoptosis, oocyte maturation, and the underlying molecular mechanisms of these processes in mouse primary granulosa cells, extending its functions beyond its primary role in several biological regulatory processes [6,21,22,23,24]. Moreover, we also explored the novel signaling pathway by which orexin-A regulates the proliferation and apoptosis of mouse GCs.

We first confirmed the mRNA expression of OXA and OX1R in primary granulosa cells of three- and six-week-old female mice. The results demonstrated that the mRNA expression levels of OXA and OX1R were significantly higher in GCs of six-week-old mice than in those of the three-week-old mice, suggesting that OXA plays a key regulatory role during different developmental stages of mouse GCs. OXA selectively binds to its specific receptor, OX1R, to perform all biological functions; therefore, we disrupted the expression of OX1R by siRNA transfection in mouse GCs to explore novel regulatory functions of OXA in GCs. The Western blot and RT-qPCR analysis showed that the expression of OX1R at the mRNA and protein levels was significantly inhibited by siOX1R transfection in mouse GCs.

Proliferation and apoptosis are naturally occurring physiological processes during the growth and development of follicles in the ovary [25,26,27]. The instructive interaction between the survival signals and apoptosis is crucial in the growth of follicles and decides the fate of follicles for ovulation or atresia [28]. GCs apoptosis plays a vital role in the induction of atresia in ovarian follicles [8,29]. Therefore, granulosa cells are crucial for atresia, follicular growth, oocyte maturation, maintenance of pregnancy after ovulation, and the estrous cycle [6,8,30]. Orexin-A’s ability in the regulation of cellular apoptosis has been reported [31]. In this regard, the role of the OXA–OX1R signaling in regulating mouse GCs has not been investigated. In this study, flow cytometry was used to assess the rate of apoptosis after OX1R knockdown. The percentage of apoptosis in siOX1R-transfected GCs groups was observed to be significantly higher than in the negative control groups, indicating that OXA plays an important role in preventing mouse GCs apoptosis. This is the first study to reveal that OXA via OX1R protects GCs apoptosis and, therefore, it is an antiapoptotic agent in mouse primary GCs.

To further explore the underlying mechanism of OXA-mediated apoptosis, the mRNA and protein expression of apoptosis-related genes was analyzed in mouse GCs. The knockdown of OX1R significantly upregulated the expression of Bax and caspase-3 through downregulation of the expression of Bcl-2 at the mRNA and protein levels. Moreover, the protein expression level of TNF-α was also upregulated following the OX1R knockdown. The Bcl-2 family participates in the mitochondrion-mediated apoptotic pathway, and this is regarded as a primary factor in regulating germ cells apoptosis in females [32,33,34]. The overexpression of Bax accelerates cellular apoptosis [35]. Caspase-3 is a key factor in increasing the apoptosis rate in all kinds of cells, which initiates a cascade of caspases to induce apoptosis in cells [36,37]. Our results suggest that the OXA–OX1R signaling protects mouse GCs apoptosis through modulating the caspase-3-dependent apoptotic pathway.

Proliferation is a critical process in the physiology of the ovary and takes part in the growth of oocytes and folliculogenesis [38]. Granulosa cells change their physiological and morphological properties during follicular formation, and these changes are connected to cell proliferation. Abnormal folliculogenesis can cause ovarian pathologies like cancer, infertility, and polycystic ovarian syndrome [39]. The results of this study clearly show that siRNA-transfected mouse GCs had a lower proliferation capacity in comparison with the negative control groups, suggesting the pro-proliferative function of OXA in mouse granulosa cells. Moreover, the expression of PCNA, a proliferation marker [40], was significantly decreased at the protein and mRNA levels after the OX1R downregulation, reinforcing our proliferation assay results. Our findings agree with a previous report showing that OXA treatment stimulated the proliferation in pancreatic cancer cells by inhibiting apoptosis through the Akt/mTOR signaling pathway [41].

Transition of the cell cycle from one phase to the next varies in different cells. The G1/S checkpoint is very important for regulating cell proliferation by transmitting and integrating molecules into the nucleus through intracellular and extracellular signals [42]. Cell proliferation is generally controlled at the G1 phase in the cell division cycle. In this study, the cell cycle assay results showed that downregulation of OX1R resulted in the S-phase arrest in the cell cycle of mouse GCs, which is the phase of DNA replication. Our results also showed the upregulated expression of the cell cycle inhibitor gene, P21, in mouse GCs following the downregulation of OX1R. As cell cycle arrest is a significant stopping point of cell division activities or cell duplication, we infer that OX1R knockdown promotes GCs apoptosis and inhibits proliferation by regulating the cell cycle.

The expression of the AKT and ERK proteins and their key role in the regulation of apoptosis, proliferation, differentiation of all types of cells have been reported, and other important physiological functions are also the result of phosphorylation of these transcription factors [43,44]. Granulosa cell proliferation is a crucial step in the development of follicles. Several previous research reports have demonstrated the involvement of the AKT and ERK signaling pathways [45]. The molecular mechanism by which orexin-A/OX1R signaling regulates proliferation and apoptosis of mouse GCs is not clear. Therefore, we further investigated how OXA influences GCs proliferation and apoptosis. Our study results showed that downregulation of OX1R resulted in the inhibition of phosphorylation levels of the ERK1/2 and AKT proteins, thereby suppressing proliferation and promoting apoptosis in mouse GCs. Consistent with our findings, previous reports showed that orexin-A regulates the ERK1/2 and AKT pathway-mediated proliferation and apoptosis in hepatocytes [31] and 3T3-L1 preadipocytes [46]. Therefore, this study suggests that orexin-A is a positive regulator of the AKT/ERK1/2 signaling pathways and promotes proliferation and inhibits apoptosis of mouse GCs through the AKT/ERK1/2 signaling pathway.

Bone morphogenetic protein 15 (BMP15) and growth differentiation factor 9 (GDF9) are secreted from oocytes [47]. These factors are involved in the regulation of ovarian functions [3,48] and play an essential role in the maturation and growth of primary follicles. In this study, the mRNA expression of BMP15 was downregulated while GDF9 was upregulated in OX1R-silenced mouse GCs. The inhibited expression of BMP15 leads to the disruption of CC expansion, which causes impaired ovulation [49]. Since the functions of OXA in reproductive cells have recently been identified, the observed dysregulation of BMP15 and GDF9 can be the molecular mechanisms by which OXA functions to maintain proper proliferation of granulosa cells and effective fertility in females.

## 5. Conclusions

In conclusion, this study demonstrated the expression of orexin-A (OXA) and orexin receptor 1 (OX1R) in mouse primary granulosa cells (GCs). The siRNA-mediated inhibition of OX1R promoted apoptosis and inhibited proliferation in mouse GCs by triggering the S-phase arrest in the cell cycle progression via the novel signaling pathway (AKT/ERK1/2 signaling pathway). Moreover, OX1R downregulation altered the expression of oocyte-secreted factors BMP15 and GDF9. These findings provide extensive functional evidence of orexin-A and its receptor in the mouse primary granulosa cells development and functions. Therefore, orexin-A can be used as a therapeutic target for the treatment of female infertility.

## Figures and Tables

**Table 1:** The sequences of NC and siRNAs used in this study.

siRNAs	Sense	Antisense
NC	UUCUCCGAACGUGUCACGUTT	ACGUGACACGUUCGGAGAATT
siOX1R-A	GGAGGAAGACGGCUAAGAUTT	AUCUUAGCCGUCUUCCUCCTT
siOX1R-B	GGCUUUGUGCAAGGUCAUUTT	AAUGACCUUGCACAAAGCCTT
siOX1R-C	GCCACCCACUGUUGUUCAATT	UUGAACAACAGUGGGUGGCTT

**Table 2:** The sequences of primers used for RT-qPCR.

Gene Symbol	Primer Sequences	Temp. (℃)	Product (bp)
OXA-F	GCGCAGAGCTAGAGCCACAT	59	192
OXA-R	TGCTAAAGCGGTGGTAGTTACG		
OX1R-F	GCTAGTGTACGCCAACAGTG	53	216
OX1R-R	GACAGCACGGTAGTGACGG		
P21-F	AACTGACTGCTCCCCTGTCTA	57	108
P21-R	CTCTATGGTTACCGCCTCCTC		
PCNA-F	GTGGATAAAGAAGAGGAGGCG	58	111
PCNA-R	TGTAGGAGACAGTGGAGTGGC		
Bax-F	GCCTCCTCTCCTACTTCGG	55	187
Bax-R	AAAAATGCCTTTCCCCTTC		
Bcl-2-F	TCTCTCGTCGCTACCGTCG	58	123
Bcl-2-R	CCCAGTTCACCCCATCCCT		
GDF9-F	CGGCTCCATCGCTTACAAA	54	187
GDF9-R	CTTCCCCCGCTCACACAGT		
BMP15-F	GAAAATGGTGAGGCTGGTAA	59	152
BMP15-R	GATGAAGTTGATGGCGGTAA		
β-Actin-F	CACGATGGAGGGGCCGGACTCATC	55	241
β-Actin-R	TAAAGACCTCTATGCCAACACAGT		

## Data Availability

Data is contained within the article.

## References

[1] Reis F.M., Cobellis L., Luisi S., Driul L., Florio P., Faletti A., Petraglia F. (2000). Paracrine/autocrine control of female reproduction. Gynecol. Endocrinol..

[2] Salustri A., Camaioni A., D’Alessandris C. (1996). Endocrine and paracrine regulation of cumulus expansion. Zygote.

[3] Emori C., Sugiura K. (2014). Role of oocyte-derived paracrine factors in follicular development. Anim. Sci. J..

[4] Sugiura K., Pendola F.L., Eppig J.J. (2005). Oocyte control of metabolic cooperativity between oocytes and companion granulosa cells: Energy metabolism. Dev. Biol..

[5] Wang S., Liu B., Liu W., Xiao Y., Zhang H., Yang L. (2017). The effects of melatonin on bovine uniparental embryos development in vitro and the hormone secretion of COCs. PeerJ.

[6] Li R., Albertini D.F. (2013). The road to maturation: Somatic cell interaction and self-organization of the mammalian oocyte. Nat. Rev. Mol. Cell Biol..

[7] Tiwari M., Prasad S., Tripathi A., Pandey A.N., Ali I., Singh A.K., Shrivastav T.G., Chaube S.K. (2015). Apoptosis in mammalian oocytes: A review. Apoptosis.

[8] Choi J., Jo M., Lee E., Choi D. (2011). Induction of apoptotic cell death via accumulation of autophagosomes in rat granulosa cells. Fertil. Steril..

[9] de Lecea L., Kilduff T.S., Peyron C., Gao X.-B., Foye P.E., Danielson P.E., Fukuhara C., Battenberg E.L.F., Gautvik V.T., Bartlett F.S. (1998). The hypocretins: Hypothalamus-specific peptides with neuroexcitatory activity. Proc. Natl. Acad. Sci. USA.

[10] Sakurai T., Amemiya A., Ishii M., Matsuzaki I., Chemelli R.M., Tanaka H., Williams S.C., Richardson J.A., Kozlowski G.P., Wilson S. (1998). Orexins and Orexin Receptors: A Family of Hypothalamic Neuropeptides and G Protein-Coupled Receptors that Regulate Feeding Behavior. Cell.

[11] Liguori G., Tafuri S., Miyoshi C., Yanagisawa M., Squillacioti C., De Pasquale V., Mirabella N., Vittoria A., Costagliola A. (2018). Localization of orexin B and orexin-2 receptor in the rat epididymis. Acta Histochem..

[12] Sakurai T. (2014). The role of orexin in motivated behaviours. Nat. Rev. Neurosci..

[13] Xu T.R., Yang Y., Ward R., Gao L., Liu Y. (2013). Orexin receptors: Multi-functional therapeutic targets for sleeping disorders, eating disorders, drug addiction, cancers and other physiological disorders. Cell. Signal..

[14] Heinonen M.V., Purhonen A.K., Mäkelä K.A., Herzig K.H. (2008). Functions of orexins in peripheral tissues. Acta Physiol..

[15] Silveyra P., Cataldi N.I., Lux-Lantos V.A., Libertun C. (2010). Role of orexins in the hypothalamic-pituitary-ovarian relationships. Acta Physiol..

[16] Silveyra P., Catalano P.N., Lux-Lantos V., Libertun C. (2007). Impact of proestrous milieu on expression of orexin receptors and preproorexin in rat hypothalamus and hypophysis: Actions of Cetrorelix and Nembutal. Am. J. Physiol. Metab..

[17] Cataldi N.I., Lux-Lantos V.A.R., Libertun C. (2012). Effects of orexins A and B on expression of orexin receptors and progesterone release in luteal and granulosa ovarian cells. Regul. Pept..

[18] Kiezun M., Smolinska N., Dobrzyn K., Szeszko K., Rytelewska E., Kaminski T. (2016). The effect of orexin A on CYP17A1 and CYP19A3 expression and on oestradiol, oestrone and testosterone secretion in the porcine uterus during early pregnancy and the oestrous cycle. Theriogenology.

[19] Rytelewska E., Kisielewska K., Gudelska M., Kiezun M., Dobrzyn K., Bors K., Wyrebek J., Kaminska B., Kaminski T., Smolinska N. (2019). The effect of orexin a on the StAR, CYP11A1 and HSD3B1 gene expression, as well as progesterone and androstenedione secretion in the porcine uterus during early pregnancy and the oestrous cycle. Theriogenology.

[20] Gao Y., Wen H., Wang C., Li Q. (2013). SMAD7 antagonizes key TGFβ superfamily signaling in mouse granulosa cells in vitro. Reproduction.

[21] Butterick T.A., Nixon J.P., Billington C.J., Kotz C.M. (2012). Orexin A decreases lipid peroxidation and apoptosis in a novel hypothalamic cell model. Neurosci. Lett..

[22] Hoyer D., Jacobson L.H. (2013). Orexin in sleep, addiction and more: Is the perfect insomnia drug at hand?. Neuropeptides.

[23] Inutsuka A., Yamanaka A. (2013). The physiological role of orexin/hypocretin neurons in the regulation of sleep/wakefulness and neuroendocrine functions. Front. Endocrinol..

[24] Kodadek T., Cai D. (2010). Chemistry and biology of orexin signaling. Mol. Biosyst..

[25] Iijima K., Jiang J.Y., Shimizu T., Sasada H., Sato E. (2005). Acceleration of follicular development by administration of vascular endothelial growth factor in cycling female rats. J. Reprod. Dev..

[26] Quirk S.M., Cowan R.G., Harman R.M., Hu C.L., Porter D.A. (2004). Ovarian follicular growth and atresia: The relationship between cell proliferation and survival. J. Anim. Sci..

[27] Sargent K.M., Lu N., Clopton D.T., Pohlmeier W.E., Brauer V.M., Ferrara N., Silversides D.W., Cupp A.S. (2015). Loss of vascular endothelial growth factor a (VEGFA) isoforms in granulosa cells using pDmrt-1-cre or Amhr2-Cre reduces fertility by arresting follicular development and by reducing litter size in female mice. PLoS ONE.

[28] Antti K., Aaron J.W.H. (1997). Regulation of ovarian follicle atresia. Annu. Rev. Physiol..

[29] Jiang J., Cheung C.K.M., Wang Y., Tsang B.K. (2003). Regulation of cell death and cell survival gene expression during ovarian follicular development and atresia. Front. Biosci..

[30] Arosh J.A., Banu S.K., Chapdelaine P., Madore E., Sirois J., Fortier A.M. (2004). Prostaglandin biosynthesis, transport, and signaling in corpus luteum: A basis for autoregulation of luteal function. Endocrinology.

[31] Ju S.J., Zhao Y., Chang X., Guo L. (2014). Orexin a protects cells from apoptosis by regulating FoxO1 and mTORC1 through the OX1R/PI3K/AKT signaling pathway in hepatocytes. Int. J. Mol. Med..

[32] Espino J., Bejarano I., Ortiz A., Lozano G.M., García J.F., Pariente J.A., Rodríguez A.B. (2010). Melatonin as a potential tool against oxidative damage and apoptosis in ejaculated human spermatozoa. Fertil. Steril..

[33] Kim M.-R., Tilly J.L. (2004). Current concepts in Bcl-2 family member regulation of female germ cell development and survival. Biochim. Biophys. Acta (BBA)—Bioenerg..

[34] Radogna F., Albertini M.C., de Nicola M., Diederich M., Bejarano I., Ghibelli L. (2015). Melatonin promotes Bax sequestration to mitochondria reducing cell susceptibility to apoptosis via the lipoxygenase metabolite 5-hydroxyeicosatetraenoic acid. Mitochondrion.

[35] Oltval Z.N., Milliman C.L., Korsmeyer S.J. (1993). Bcl-2 heterodimerizes in vivo with a conserved homolog, Bax, that accelerates programed cell death. Cell.

[36] Cai K., Hua G., Ahmad S., Liang A., Han L., Wu C., Yang F., Yang L. (2011). Action Mechanism of Inhibin α-Subunit on the Development of Sertoli Cells and First Wave of Spermatogenesis in Mice. PLoS ONE.

[37] Han L., Wu C., Riaz H., Bai L., Chen J., Zhen Y., Guo A., Yang L. (2013). Characterization of the Mechanism of Inhibin α-Subunit Gene in Mouse Anterior Pituitary Cells by RNA Interference. PLoS ONE.

[38] Hussein M.R. (2005). Apoptosis in the ovary: Molecular mechanisms. Hum. Reprod. Update.

[39] Wu J., Emery B.R., Carrell T.D. (2001). In vitro growth, maturation, fertilization, and embryonic development of oocytes from porcine preantral follicles. Biol. Reprod..

[40] Iatropoulos M.J., Williams G.M. (1996). Proliferation markers. Exp. Toxicol. Pathol..

[41] Suo L., Chang X., Zhao Y. (2018). The orexin-A-regulated Akt/mTOR pathway promotes cell proliferation through inhibiting apoptosis in pancreatic cancer cells. Front. Endocrinol. (Lausanne).

[42] Skotheim J.M., di Talia S., Siggia E.D., Cross F.R. (2008). Positive feedback of G1 cyclins ensures coherent cell cycle entry. Nature.

[43] Chang F., Steelman L.S., Lee J.T., Shelton J.G., Navolanic P.M., Blalock W.L., Franklin R.A., McCubrey J. (2003). Signal transduction mediated by the Ras/Raf/MEK/ERK pathway from cytokine receptors to transcription factors: Potential targeting for therapeutic intervention. Leukemia.

[44] Lefloch R., Pouysségur J., Lenormand P. (2008). Single and Combined Silencing of ERK1 and ERK2 Reveals Their Positive Contribution to Growth Signaling Depending on Their Expression Levels. Mol. Cell. Biol..

[45] Ryan K.E., Casey S.M., Canthy M.J., Crowe M.A., Martin F., Evans A.C.O. (2007). Akt and Erk signal transduction pathways are early markers of differentiation in dominant and subordinate ovarian follicles in cattle. Reproduction.

[46] Skrzypski M., Kaczmarek P., Le T., Wojciechowicz T., Pruszynska-Oszmalek E., Szczepankiewicz D., Sassek M., Arafat A., Wiedenmann B., Nowak K.W. (2012). Effects of orexin A on proliferation, survival, apoptosis and differentiation of 3T3-L1 preadipocytes into mature adipocytes. FEBS Lett..

[47] Paulini F., Melo E.O. (2010). The Role of Oocyte-Secreted Factors GDF9 and BMP15 in Follicular Development and Oogenesis. Reprod. Domest. Anim..

[48] Diaz F.J., Wigglesworth K., Eppig J.J. (2007). Oocytes determine cumulus cell lineage in mouse ovarian follicles. J. Cell Sci..

[49] Yoshino O., McMahon H.E., Sharma S., Shimasaki S. (2006). A unique preovulatory expression pattern plays a key role in the physiological functions of BMP-15 in the mouse. Proc. Natl. Acad. Sci. USA.

